# Non-operative Management of a Rare Segmental Clavicle Fracture in an Adolescent Patient: A Case Report

**DOI:** 10.7759/cureus.42508

**Published:** 2023-07-26

**Authors:** Peter Harimtepathip, George Puneky, Justin Lomax, Cory Bryan

**Affiliations:** 1 Department of Orthopedics, Augusta University Medical College of Georgia, Augusta, USA

**Keywords:** segmental clavicle fracture, pediatric clavicle fracture, nonoperative treatment clavicle fracture, pediatric orthopaedics, adolescent clavicle fracture

## Abstract

A 12-year-old Caucasian male presented to the clinic with a displaced, segmental left clavicle fracture involving the distal clavicle after falling from a zip line. He was treated non-operatively in a sling and returned to normal activities without restrictions after three months. At one year, the patient was able to maintain his pre-injury baseline function without limitations of his left shoulder. While no clear guidelines for operative treatment of segmental clavicle fractures in the adolescent population exist in the current literature, this report illustrates an excellent patient outcome following conservative therapy of a segmental clavicle fracture involving the middle one-third and distal clavicle in a young adolescent with open physis.

## Introduction

Clavicle fractures are common pediatric injuries in which the majority are treated non-operatively [1]. The annual incidence of clavicle fractures in the adolescent population is between 29 and 64 per 100,000 [2]. These fractures are often caused by direct injury after a fall onto the shoulder resulting in a compressive force produced by body weight, impact speed, and force direction through the clavicle [3]. Increased body weight and impact speeds seen across various sports injuries may explain the age and gender differences observed between displaced and nondisplaced midshaft clavicle fractures [1,3,4]. Despite growing literature regarding the surgical management of pediatric clavicle fractures, very little has been reported concerning the conservative treatment of segmental fractures of the clavicle. This is likely due to the rare presentation of these fracture patterns [5]. Nevertheless, the rate of operative fixation of displaced clavicle fractures in pediatric patients has steadily increased over the past century from 1.6% to 24%, mimicking the trends seen in skeletally mature patients [6,7]. The relative indications for surgical management of pediatric and adolescent clavicle fractures include initial shortening, comminution, severe displacement, open injuries, skin compromise, and associated neurovascular injuries [3]. However, most pediatric clavicle fractures are treated conservatively due to reports of excellent healing and remodeling potential [3]. The optimal treatment decision for displaced, comminuted clavicle fractures can become difficult when assessing young adolescent patients. The youthful healing and remodeling potential must be weighed against the potential morbidity of displaced, comminuted fractures [8,9]. Moreover, extrapolation of superior outcomes of operatively managed clavicles in older adolescents and young adults can lead one to question the non-operative management of high-energy fracture patterns in these age groups [10]. The current report describes the non-operative management of a displaced, segmental clavicle fracture with fragments spanning both the midshaft and distal clavicle. The patient and respective guardians were informed that data from the case would be submitted for publication and provided consent.

## Case presentation

**Figure 1:**
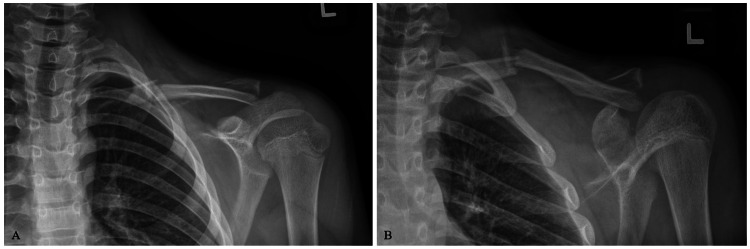
Injury radiographs, upright anteroposterior and Zanca views (A-B), demonstrating displaced, segmental clavicle fracture.

A 12-year-old Caucasian male with no significant past medical history presented to the clinic with a closed left clavicle fracture after falling from a zip line 12 days prior. A physical exam showed a deformity over the left clavicle without appreciable skin tenting or neurovascular injury. Initial radiographs were obtained demonstrating a displaced, segmental clavicle fracture involving the middle one-third and distal clavicle, lateral to the coracoid (Figure 1). The acromioclavicular interval and coracoclavicular intervals were maintained on injury radiographs.

**Figure 2:**
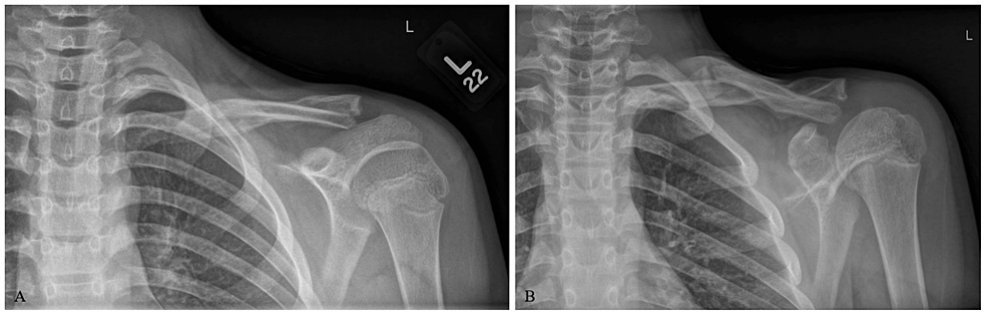
Six-week post-injury radiographs, upright anteroposterior and Zanca views (A-B), demonstrating interval healing and callus formation at the fracture sites.

**Figure 3:**
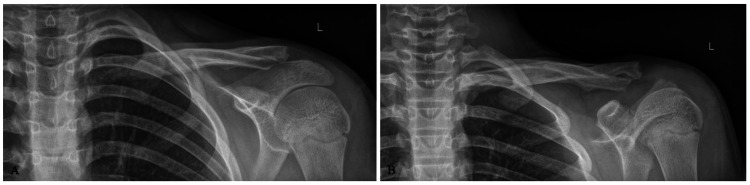
Three-month post-injury radiographs, upright anteroposterior and Zanca views (A-B), demonstrating continued fracture healing and early remodeling.

**Figure 4:**
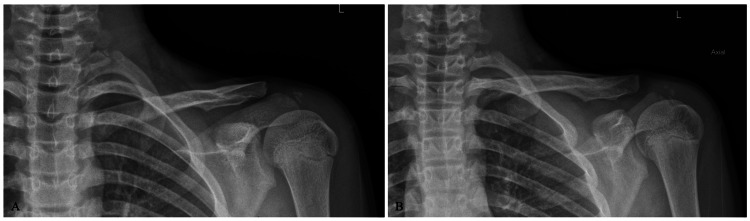
One-year post-injury radiographs, upright anteroposterior and Zanca views (A-B), demonstrating progressive remodeling of the initial segmental clavicle fracture.

The patient was treated non-operatively in a sling and instructed to remain non-weight bearing to the left upper extremity. Radiographs were obtained six weeks post-injury demonstrating interval callus formation of the fracture sites (Figure 2), and the patient was allowed to progress activities as tolerated with restrictions to avoid high-fall-risk activities. At three months post-injury, the patient was cleared for full activity without restrictions (Figure 3). At one year, the patient denied functional limitations or aesthetic complaints related to his left shoulder. Follow-up radiographs obtained at this time demonstrated notable remodeling of the left clavicle fracture sites (Figure 4).

## Discussion

Unlike in adults, the risk of nonunion and symptomatic malunion in children is uncommon due to their ability to heal and remodel clavicle fractures; thus, these patients have traditionally been treated conservatively. A study completed in 2013 evaluated pain, aesthetic appearance, treatment satisfaction, and functional outcomes of 16 patients (mean age 12.2 years) with mid-diaphyseal clavicle fracture malunions after non-operative treatment. All patients in the given study presented with an initial fracture displacement greater than 2 centimeters and were followed for two years. The authors concluded that radiographic malunion in adolescents does not result in clinically meaningful loss of shoulder motion or strength [11]. A retrospective review of nine high-volume pediatric hospitals from 2006 to 2016 identified only 25 patients between the ages of 10 and 19 years (mean 14.6 years) with clavicle nonunion following conservative therapy. All nonunions were subsequently treated with surgery resulting in good outcomes [12,13]. Two retrospective reviews reported the rate of symptomatic malunion in the pediatric population varies from 2 to 20%, while additional studies have shown no significant impact on patient reported outcomes in the setting of radiographic malunion [9,11,12,14]. A recent study looking at functional outcomes in 20 patients with midshaft clavicle fractures age 12-18 years at the time of injury showed no statistical difference between operative and non-operative treatment over 18 months of follow up [12,15]. Prophylactic surgery to decrease the risk of symptomatic malunion is not advantageous relative to an initial trial of conservative therapy when analyzing cost and patient reported outcomes. A study of 16 pediatric patients treated with delayed surgery for malunion, nonunion, or impending nonunion reported good functional outcomes and 100% return to sports [16]. Using the 2013 Medicare reimbursement model, the total costs saving of non-operative management is $11,60.56 [17].

Although non-operative management has traditionally been the treatment of choice for pediatric clavicle fractures, there has been a recent increase in operative management. As patients enter adolescence they become more active than adults, gradually lose their remodeling potential, and may experience greater functional impairment from residual disability. The initial displacement of a clavicle fracture is an important factor in treatment decision. Initial fracture shortening of >20mm is associated with nonunion and unsatisfactory results [8]. A Canadian study reviewed 132 patients between age 16 and 60 years with displaced midshaft clavicle fractures in which 67 were treated operatively and 65 were treated non-operatively. They determined that the operative group experienced better functional outcomes at one year, with a mean time to radiographic union of 28.4 weeks in the non-operative group and 16.4 weeks in the operative group. Additionally, non-operative treatment resulted in seven unions and nine malunions requiring further treatment, while the operative group noted two nonunions and zero malunions. The authors concluded that patients treated operatively are more likely to be satisfied with their appearance and shoulder function shoulder in general [10]. A retrospective study from 2010 investigated 43 closed midshaft clavicle fractures (25 non-operative, 17 operative). The operative group underwent plate fixation for displaced midshaft clavicle fractures resulting in a shorter time to union with low complication rates, while the non-operative group resulted in a 20% symptomatic malunion rate [18]. Limited literature exists comparing patient outcomes related to surgical versus conservative therapy of pediatric clavicle fractures. This has resulted in the lack of clear operative criteria by which surgeons can apply when treating these injuries in the young population.

A disparity exists amongst surgeons regarding the treatment of adolescent clavicle fractures as shown by a survey of 302 members of the Pediatric Orthopaedic Society of North America (POSNA). Surgeons were nearly unanimously in favor of non-operative treatment in younger patients. However, regarding isolated segmental clavicle fractures, treatment preferences were split between operative and non-operative management with surgeons more likely to operate on adolescents age 16-19 years than age 12-15 years. Additionally, surgeons with less than five years of experience were more likely to choose operative treatment [2]. A retrospective study looked at 10 pediatric distal clavicle fractures from children age 5-11 years (mean age seven). Nine patients were treated conservatively (three displaced oblique fractures, three displaced transverse fractures, two nondisplaced transverse fractures, and one comminuted fracture). One 11-year-old female with a comminuted distal clavicle fracture was treated with open reduction and Kirschner wire fixation. All patients reported good functional outcomes, although the authors recommended magnetic resonance imaging (MRI) for significantly displaced fractures to assess the degree of periosteum detachment [19]. Other studies have described symptomatic deformities following significantly displaced distal clavicle physeal fractures, suggesting benefit of early fixation [20].

## Conclusions

Clavicle fractures in the adolescent population have no clear consensus on treatment, particularly when considering the 12-15-year-old age group. Currently, no literature supports the age range at which surgeons should adopt more aggressive treatment options related to fracture characteristics. This report demonstrates that a segmental clavicle fracture involving the middle one-third and distal clavicle in a young adolescent with open physes may be managed conservatively with good outcomes.

## References

[1] Nordqvist A, Petersson C (1994). The incidence of fractures of the clavicle. Clin Orthop Relat Res.

[2] Carry PM, Koonce R, Pan Z, Polousky JD (2011). A survey of physician opinion: adolescent midshaft clavicle fracture treatment preferences among POSNA members. J Pediatr Orthop.

[3] Neer C (1984). Fractures of the Clavicle. Rockwood and Green's Fractures in Adults.

[4] Stanley D, Trowbridge EA, Norris SH (1988). The mechanism of clavicular fracture. A clinical and biomechanical analysis. J Bone Joint Surg Br.

[5] Throckmorton T, Kuhn JE (2007). Fractures of the medial end of the clavicle. J Shoulder Elbow Surg.

[6] Ellis HB, Li Y, Bae DS (2020). Descriptive epidemiology of adolescent clavicle fractures: results from the FACTS (function after adolescent clavicle trauma and surgery) prospective, multicenter cohort study. Orthop J Sports Med.

[7] Yang S, Werner BC, Gwathmey FW Jr (2015). Treatment trends in adolescent clavicle fractures. J Pediatr Orthop.

[8] Hill JM, McGuire MH, Crosby LA (1997). Closed treatment of displaced middle-third fractures of the clavicle gives poor results. J Bone Joint Surg Br.

[9] Robinson L, Gargoum R, Auer R, Nyland J, Chan G (2015). Sports participation and radiographic findings of adolescents treated nonoperatively for displaced clavicle fractures. Injury.

[10] Canadian Orthopaedic Trauma Society (2007). Nonoperative treatment compared with plate fixation of displaced midshaft clavicular fractures. A multicenter, randomized clinical trial. J Bone Joint Surg Am.

[11] Bae DS, Shah AS, Kalish LA, Kwon JY, Waters PM (2013). Shoulder motion, strength, and functional outcomes in children with established malunion of the clavicle. J Pediatr Orthop.

[12] Vargas-Vila MA, Mehlman CT, Pennock AT (2019). The community orthopaedic surgeon taking trauma call: pediatric midshaft clavicle fracture pearls and pitfalls. J Orthop Trauma.

[13] Pennock AT, Edmonds EW, Bae DS (2018). Adolescent clavicle nonunions: potential risk factors and surgical management. J Shoulder Elbow Surg.

[14] Schulz J, Moor M, Roocroft J, Bastrom TP, Pennock AT (2013). Functional and radiographic outcomes of nonoperative treatment of displaced adolescent clavicle fractures. J Bone Joint Surg Am.

[15] Herzog MM, Whitesell RC, Mac LM (2017). Functional outcomes following non-operative versus operative treatment of clavicle fractures in adolescents. J Child Orthop.

[16] Carsen S, Bae DS, Kocher MS, Waters PM, Donohue K, Heyworth BE (2015). Outcomes of operatively treated non-unions and symptomatic mal-unions of adolescent diaphyseal clavicle fractures. Orthop J Sports Med.

[17] Walton B, Meijer K, Melancon K, Hartman M (2015). A cost analysis of internal fixation versus nonoperative treatment in adult midshaft clavicle fractures using multiple randomized controlled trials. J Orthop Trauma.

[18] Vander Have KL, Perdue AM, Caird MS, Farley FA (2010). Operative versus nonoperative treatment of midshaft clavicle fractures in adolescents. J Pediatr Orthop.

[19] Labronici PJ, da Silva RR Jr, Franco MV, Labronici GJ, Pires RE, Franco JS (2016). Distal clavicle fractures in children. Rev Bras Ortop.

[20] Ogden J (1984). Distal clavicular physeal injury. Clin Orthop Relat Res.

